# Metal recovery from spent lithium-ion batteries via two-step bioleaching using adapted chemolithotrophs from an acidic mine pit lake

**DOI:** 10.3389/fmicb.2024.1347072

**Published:** 2024-01-30

**Authors:** Lalropuia Lalropuia, Jiri Kucera, Wadih Y. Rassy, Eva Pakostova, Dominik Schild, Martin Mandl, Klemens Kremser, Georg M. Guebitz

**Affiliations:** ^1^K1-MET GmbH, Linz, Austria; ^2^Department of Biochemistry, Faculty of Science, Masaryk University, Brno, Czechia; ^3^Department of Science and Technology, Institute of Biotechnology, IMC University of Applied Sciences, Krems, Austria; ^4^Faculty of Technical Chemistry, TU Wien, Vienna, Austria; ^5^Department of Earth and Environmental Sciences, University of Waterloo, Waterloo, ON, Canada; ^6^Department of Agrobiotechnology, IFA-Tulln, Institute of Environmental Biotechnology, University of Natural Resources and Life Sciences Vienna BOKU, Tulln an der Donau, Austria

**Keywords:** acidic mine pit lake, bacterial adaptation, bioleaching, black mass, lithium-ion batteries, metal recovery, microbial enrichment

## Abstract

The demand for lithium-ion batteries (LIBs) has dramatically increased in recent years due to their application in various electronic devices and electric vehicles (EVs). Great amount of LIB waste is generated, most of which ends up in landfills. LIB wastes contain substantial amounts of critical metals (such as Li, Co, Ni, Mn, and Cu) and can therefore serve as valuable secondary sources of these metals. Metal recovery from the black mass (shredded spent LIBs) can be achieved via bioleaching, a microbiology-based technology that is considered to be environmentally friendly, due to its lower costs and energy consumption compared to conventional pyrometallurgy or hydrometallurgy. However, the growth and metabolism of bioleaching microorganisms can be inhibited by dissolved metals. In this study, the indigenous acidophilic chemolithotrophs in a sediment from a highly acidic and metal-contaminated mine pit lake were enriched in a selective medium containing iron, sulfur, or both electron donors. The enriched culture with the highest growth and oxidation rate and the lowest microbial diversity (dominated by *Acidithiobacillus* and *Alicyclobacillus* spp. utilizing both electron donors) was then gradually adapted to increasing concentrations of Li^+^, Co^2+^, Ni^2+^, Mn^2+^, and Cu^2+^. Finally, up to 100% recovery rates of Li, Co, Ni, Mn, and Al were achieved via two-step bioleaching using the adapted culture, resulting in more effective metal extraction compared to bioleaching with a non-adapted culture and abiotic control.

## 1 Introduction

Due to their high energy density and longevity, lithium-ion batteries (LIBs) dominate the battery market, with their use ranging from portable electronic devices to electric vehicles (EV). LIBs contain an anode (alloys, carbon, silicon, and transition metal oxides), lithium metal oxide cathode, and liquid electrolyte. The most common types of LIB cathode materials include Lithium Cobalt Oxide (LCO), Lithium Nickel Manganese Cobalt Oxide (NMC), and Lithium Iron Phosphate (LFP) (Boyden et al., 2016). The current increasing demand for LIBs has been greatly influenced by the ongoing transition from combustion engine vehicles to EVs. This is a result of primarily developed countries (the USA, Japan, and the EU) taking new initiatives to reduce carbon emissions and move towards green energy (Kim et al., 2012). It is predicted that by 2050, 50% of the global vehicle production will be EVs (Sonoc and Jeswiet, 2014). Around 200,000 tons of waste has been estimated to be generated in 2020 from LIB cathodes alone (Ali et al., 2021), and the rising LIB production and use will result in increasing amounts of waste generated. Most of the spent LIBs will end up in landfills where the present metals (as well as other components) pose a severe environmental threat; the hazardous substances have the potential to contaminate soil and groundwater and could be harmful to human health (Bankole et al., 2013).

Since spent LIBs contain high concentrations of critical metals such as Co, Ni, Mn, and Li, they can be used as an important secondary source for these metals. According to the EU Battery Regulation, 65 and 70% of Li-based batteries should be recycled in 2025 and 2030, respectively, with recycling rates of 35 and 70% for Li in 2023 and 2030, respectively, and 90 and 95% for Co, Ni and Cu in 2025 and 2030, respectively (Regulation (EU), 2023). There is tremendous financial revenue in LIB recycling, as the current value is estimated to be $860 per ton for LiMnO_4_-based batteries and approximately $8,900 per ton for LiCoO_2_-based cathodes (Wang et al., 2014). As natural resources used in LIBs are limited and the production is concentrated only in a few countries (e.g., China), risks of disruption of the supply of critical raw materials for LIBs are significant (Sun et al., 2019). Therefore, recycling spent LIBs could help mitigate the negative environmental impacts, minimize waste production, and lower the mining of primary mineral resources (Bankole et al., 2013). Current recycling processes are mainly based on pyrometallurgical, mechanical, and hydrometallurgical methods, which have many disadvantages including the production of large amounts of hazardous wastes (Boyden et al., 2016). To recover metals from spent LIB cathode materials, mainly strong inorganic acids, such as HCl, H_2_SO_4_, and HNO_3_ are used. This approach provides high metal recovery rates, but harsh chemicals have a negative environmental impact and produce hazardous wastes (Mossali et al., 2020). Recently, research on the application of bioprocesses in metal recycling has become an emerging topic. Organic acids such as citric, malic, and aspartic acids have proven to be suitable leaching agents. Almost 100% of Li and Co was recovered from LIBs using organic acids in the presence of H_2_O_2_ (Li et al., 2013a).

Bioleaching, a process in which metals are solubilized using microorganisms, could be a “green” alternative technology for recovering critical metals from spent LIBs. Bioleaching is cost-efficient and provides several advantages over conventional recycling methods, including a lower production of hazardous wastes and lower energy consumption (Hansford and Vargas, 2001). Most bioleaching microorganisms are acidophilic, thriving at low pH, and chemolithotrophic, utilizing inorganic compounds as electron donors, such as Fe^2+^ and reduced inorganic sulfur compounds (RISCs). The microorganisms can be heterotrophic, metabolizing organic substrates such as glucose, autotrophic, fixating CO_2_, or mixotrophic, using both organics and CO_2_. Among the most prominent bacterial genera are *Acidithiobacillus* (*A.*)*, Sulfobacillus, Leptospirillum* (*L.*), and many others, while *Ferroplasma*, *Acidiplasma,* and *Sulfolobus* belong to archaea. Bioleaching is primarily used to extract metals from low-grade sulfidic ores in which the Fe^2+^- and RISC-oxidizing microbes solubilize metals via the production of Fe^3+^ and H_2_SO_4_, respectively (Sajjad et al., 2019). Bioleaching has also been shown to be feasible for recycling of e-waste, such as printed circuit boards (PCBs), and other waste streams, such as metal-bearing ashes and slags. For example, 96% Cu, 73% Ni, and 93% Co were recovered from PCBs using *L. ferriphilum* and *Sulfolobus benefaciens* in a bioreactor (Hubau et al., 2020). Another study investigated the bioleaching of ashes and slags from incineration residues, reaching 100% leaching efficiency of Zn, Cu, and Mn using Fe^2+^- and RISC-oxidizing bacteria (Kremser et al., 2021). Similarly, metals from LIB waste can also be recovered using bioleaching, and the topic has been extensively researched in recent years. Roy et al. (2021a) reported recovery of 90% Ni, 82% Co, and 92% Mn from spent NMC-based LIBs using *A. ferrooxidans*. Cultures enriched from soil and mud samples in the lava tour area and tannery wastewater dissolved 62.8% of Li from LIBs in 15 days (Hartono et al., 2017). Up to 94% of Co and 60% of Li were recovered in 72 h, using *A. ferrooxidans* in three cycles with 10% (w/v) pulp density (Roy et al., 2021b). Another study reported 96% Co and Ni recovery from LIBs in EVs using a mixed culture of *A. thiooxidans* and *L. ferriphilum* (Xin et al., 2016).

Although most acidophiles show increased tolerance to dissolved metals, LIB wastes contain very high metal content, which, combined with the acid-consuming character of the materials, can inhibit the growth of acidophiles (Roy et al., 2021c). The microbes are often adapted to high metal concentrations before LIB bioleaching, especially in contact bioleaching approaches, to improve their metal resistance and leaching performance. Contact bioleaching involves the cultivation of the microbes in the presence of the LIB waste with simultaneous metal release. In contrast, a biogenic lixiviant is produced during non-contact bioleaching (Ilyas et al., 2007; Bajestani et al., 2014; Bahaloo-Horeh et al., 2018; Vera et al., 2022). In a study by Mishra et al. (2008), 65% of Co was leached from LIBs using adapted *A. ferrooxidans*. Mesophilic and thermophilic acidophiles are often found in extreme environments such as acid mine drainage or hot springs. These environments feature low pH (<3) and moderate to high temperatures, together with elevated dissolved metal concentrations (Dopson et al., 2004; Salo-Zieman et al., 2006). Mixed cultures enriched from such environments often show higher bioleaching efficiency than pure cultures (Xiang et al., 2010; Retnaningrum et al., 2021). However, there is limited information about applying cultures enriched from environmental samples in LIB bioleaching, as most studies used pure cultures or constructed consortia. In addition, reports on bioleaching of spent LIBs are generally limited to low pulp densities due to the toxicity of dissolved metals and the alkaline nature of LIBs (Alipanah et al., 2023).

This study aims to investigate the recovery of valuable metals (Li, Co, Ni, Cu, and Mn) from the black mass (BM) derived from spent LIBs using two-step bioleaching with microbial enrichment from the sediment of an acidic mine pit lake. Prior to BM bioleaching, the enriched culture was adapted to elevated metal concentrations using a gradual adaptation, and its leaching efficiency was compared to those of a non-adapted and abiotic control.

## 2 Materials and methods

### 2.1 Spent batteries

A partner company provided pre-treated BM from spent NMC-based LIBs. The pre-treatment process involved discharging, dismantling, thermal treatment, crushing, and sorting. Inductively coupled plasma mass spectrometry (ICP-MS) was used to determine the elemental composition of the BM (Table 1), after acid digestion of the BM using aqua regia (according to ÖNORM EN 13657:2002-12). The particle size of the BM sample was determined according to ISO 13320-1, using a HELOS (Sympatec GmbH, Germany) particle size distribution measurement device, resulting in d_10_ = 4.4 μm, d_50_ = 17.2 μm, and d_90_ = 55.0 μm.

**Table 1:** Metal contents in the black mass from spent LIBs of an NMC type.

Metal content (g/kg)						
Li	Co	Ni	Mn	Cu	Al	Fe
27.60	145.00	58.60	41.70	35.50	52.60	5.75

### 2.2 Sample collection and culture media

A sediment sample was collected (in sterile 100 mL tubes) in early November 2021 a few centimeters below the water surface in a shallow part of extremely acidic (pH ~ 2.6), metal-rich Lake Hromnice in the Czech Republic (49°51′02.5″N, 13°26′39.3″E) (Hrdinka et al., 2013). The indigenous acidophiles were enriched using a selective liquid medium for isolating acidophiles containing basal salts and trace elements, as described previously (Ňancucheo et al., 2016). Three types of selective media were prepared: (i) 50 mM FeSO_4_·7H_2_O for culturing Fe^2+^ oxidizers at pH 1.7 (Fe medium), (ii) 1% (w/v) elemental sulfur (S^0^) for culturing RISC oxidizers at pH 3.5 (S medium), and (iii) 50 mM FeSO_4_·7H_2_O and 1% (w/v) S^0^ for culturing Fe^2+^ and RISC oxidizers at pH 2.0 (FeS medium). The media were sterilized using 0.2 μm Nalgene™ Rapid-Flow™ filters (Thermo Fisher Scientific, United States).

### 2.3 Selective microbial enrichments

Acidophilic chemolithoautotrophs were enriched in Erlenmeyer flasks (100 mL working volume) containing 5 g lake sediment and Fe, S, or FeS medium. The enrichment in Fe medium was carried out for 14 days, and those in S and FeS media for 21 days at 30°C and agitation (150 rpm). After the incubation, the enriched cultures (10 mL) were inoculated into fresh media and further cultivated. The sediment and residual S^0^ were removed from the remaining 90 mL of the enriched cultures by centrifugation at 1500 *g* for 1 min, followed by harvesting the cells at 3428 *g* for 15 min. The cell pellets were stored at −80°C until DNA isolation.

During the enrichment process, 1 mL sample was withdrawn from each flask (daily during week one and alternate days from week two onwards). Each time, 1 mL of fresh medium was added to compensate for the volume loss. Samples were centrifuged at 1500 *g* for 1 min before the measurement of the optical density at 660 nm (OD_660_), followed by the determinations of pH, oxidation–reduction potential (ORP), SO_4_^2−^, and Fe/Fe^2+^ concentrations (prior to the two latter measurements, the samples were filtered through a 0.20 μm filter membrane).

### 2.4 Adaptation of acidophiles to elevated metal concentrations

The culture enriched in FeS medium was gradually adapted to increasing concentrations of Li^+^, Co^+2^, Ni^+2^, Mn^+2^, and Cu^2+^. Single-metal stock solutions (1 M) were prepared in ultra-pure water using LiCl, CoCl_2_, NiCl_2_, MnSO_4_, or CuSO_4_·5H_2_O. The adaptation of acidophiles was done in three subsequent stages in FeS medium containing increasing metal concentrations, corresponding to metal contents of 2.5, 5, and 10 g/L of NMC-based BM (Table 2). The adaptation was performed in Erlenmeyer flasks (100 mL working volume) at 30°C and under agitation (150 rpm). After 14 days, 10 mL of the culture was used as inoculum in the next adaptive stage. The ORP, pH, OD_660_, SO_4_^2−^, and Fe/Fe^2+^ values were measured as described in Section 2.6. The adapted culture was then used for two-step bioleaching of BM.

**Table 2:** Summary of the process used to adapt the microbial consortium to BM.

Adaptation step	Corresponding BM concentration	Metal concentration^a^				
	[g/L]			[g/L]		
		Li	Co	Ni	Mn	Cu
1st	2.5	0.08	0.45	0.41	0.21	0.22
2nd	5.0	0.21	1.46	0.53	0.42	0.32
3rd	10.0	0.43	2.92	1.06	0.84	0.65

a via addition of synthetic metal solutions as described in Section 2.4.

Mixtures of five target metals were added in three consecutive steps in metal concentrations corresponding to those in 2.5, 5.0, and 10.0 g/L NMC-based BM.

### 2.5 Two-step bioleaching

In the first step, 10% (v/v) of the adapted and non-adapted cultures were pre-cultivated in a fresh FeS medium (50 mL working volume, pH = 2.0) for 7 days. In the second step, 1% BM (w/v) was added, and bioleaching of metals was performed for another 7 days at 30°C and 150 rpm. In addition, an abiotic control was run in parallel by mixing 1% (w/v) of the BM with 50 mL of sterile FeS medium. Samples were taken before the addition of BM (day zero) and on days 2, 5, and 7 of metal bioleaching. The ORP, pH, OD, SO_4_^2−^, and Fe/Fe^2+^ values were monitored as described in Section 2.6. At the end of the experiment, cells and solid particles were removed by centrifugation at 3428 *g* for 15 min. Furthermore, the supernatant was filtered through a nylon filter of 0.45 μm pore size. The metal concentrations were determined in the filtrates by inductively coupled plasma optical emission spectroscopy (ICP-OES); see below. The dissolved metal concentrations in samples from day zero were subtracted from those determined in samples collected on days onwards. The solid residues were dried at 60°C for 48 h, ground, and analyzed using energy dispersive X-ray spectroscopy (EDS).

### 2.6 Analytical methods

All cultivations were performed in a Multitron Pro shaker (Infors HT, Switzerland). A pH electrode LE422 (Mettler Toledo, Switzerland) and ORP electrode InLab Redox (vs. Ag/AgCl; Mettler Toledo, Switzerland) connected to an S22O pH/ion meter (Mettler Toledo, Switzerland) were used to determine pH and ORP, respectively. A DR3900 spectrophotometer (Hach Lange, Austria) was used for OD_660_ measurement.

Fe^2+^/Fe concentrations were measured at 562 nm using 96-well plates and an Infinite 200 Pro M Plex Microplate Reader (Tecan, Switzerland). Fe^2+^ concentration was determined in 228 μL ferrozine solution mixed with 12 μL sample, and total Fe concentration was measured after a 20 min incubation with 45 μL of HONH_2_-HCl and 15 μL of NH_4_CH_3_CO_2_ added to the wells. Seven-point calibration was done over a 0–1 mM FeSO_4_·7H_2_O concentration range.

The SO_4_^2−^ concentration was measured using Dionex ICS-900 ion chromatography (Thermo Fisher Scientific, United States). A mixture of 8 mM Na_2_CO_3_ and 1 mM NaHCO_3_ was used as the eluent, and 60 mM H_2_SO_4_ was used as the regeneration solution. Before analysis, liquid samples were diluted using the eluent and filtered through a 0.2 μm filter into 0.5 mL vials. Nine-point calibration was done over a 1–300 g/L of SO_4_^2−^ (in the form of Na_2_SO_4_) concentration range.

Metal concentrations in leachate samples were determined using 5,110 ICP-OES with an ICP Expert Autosampler (Agilent, United States). 0.5 mL filtered sample through a 0.2 μm filter was mixed with 200 μL 69% (v/v) HNO_3_ and incubated at 60°C for 24 h. After cooling to room temperature (RT), the samples were treated in a Sonorex RK 100H ultrasonic bath (Bandelin, Germany) at 60°C for 30 min. After sonification and cooling to RT, the samples were topped up to 10 mL using ultrapure water (resulting in a 20-fold dilution). The concentrations of selected metals (Li, Co, Ni, Mn, Al, and Fe) were measured at eight wavelengths each, and two wavelengths specific for Ar and one wavelength specific for C were used as internal standards. Multi-metal standard solutions (0.5, 1, 2.5, and 5 ppm) were used for calibration, and 2% HNO_3_ was used as blank. A *t*-test (*p* < 0.05) was performed to assess the differences between leaching efficiency in the adapted culture, non-adapted culture, and control (abiotic) experiments. The metal content in solid residues was examined at the end of the leaching experiments using a TM 3030 scanning electron microscope (SEM) with an EDS detector (Hitachi, Japan).

The recovery rate of each metal was calculated using the following formula:


$$\mathrm{Recovery}\;\mathrm{rate}\;\left(\%\right)=\left[\left(C_{L}-C_{0}\right)/C_{BM}\right]*100$$

where C are metal concentrations in leachate (C_L_), in assay before BM addition (C_0_), and in BM (C_BM_).

### 2.7 DNA isolation and 16S rRNA amplicon sequencing

Total genomic DNA was extracted using the DNeasy UltraClean Microbial Kit (Qiagen, Netherlands), according to the manufacturer’s instructions. The hypervariable region V4 was amplified with unique barcoded oligonucleotides 515F and 806R, as described previously (Spiess et al., 2021). PCR amplification was performed using Platinum II Taq Hot-Start DNA Polymerase (Thermo Fisher Scientific, United States), as follows: initial DNA denaturation step at 94°C for 3 min, 35 cycles of DNA denaturation at 94°C for 45 s, annealing at 52°C for 60 s with a 50% thermal ramp, and extension at 72°C for 90 s, and a final extension step at 72°C for 10 min. The PCR products were purified using AMPure XP beads (Beckman Coulter, United States) following the manufacturer’s instructions. The Qubit 4.0 fluorometer (Thermo Fisher Scientific, United States) and FragmentAnalyzer (Advanced Analytical Technologies, United States) were then used to determine the library quantity and quality. The library was sequenced using a MiniSeq System (Illumina, United States) with a MiniSeq Mid Output Kit (300 cycles). The raw fastq reads were processed in R software (4.3.1) using the open-source package DADA2 (1.28.0) as described previously (Spiess et al., 2022). A summary of all amplicon sequence variants (ASVs) is shown in Supplementary Table S1. The dataset generated and analyzed in this work is available in the NCBI Sequence Read Archive under BioProject ID: PRJNA1045576.

## 3 Results and discussion

### 3.1 16S analysis of enriched cultures from an acidic mine pit lake

**Figure 1:**
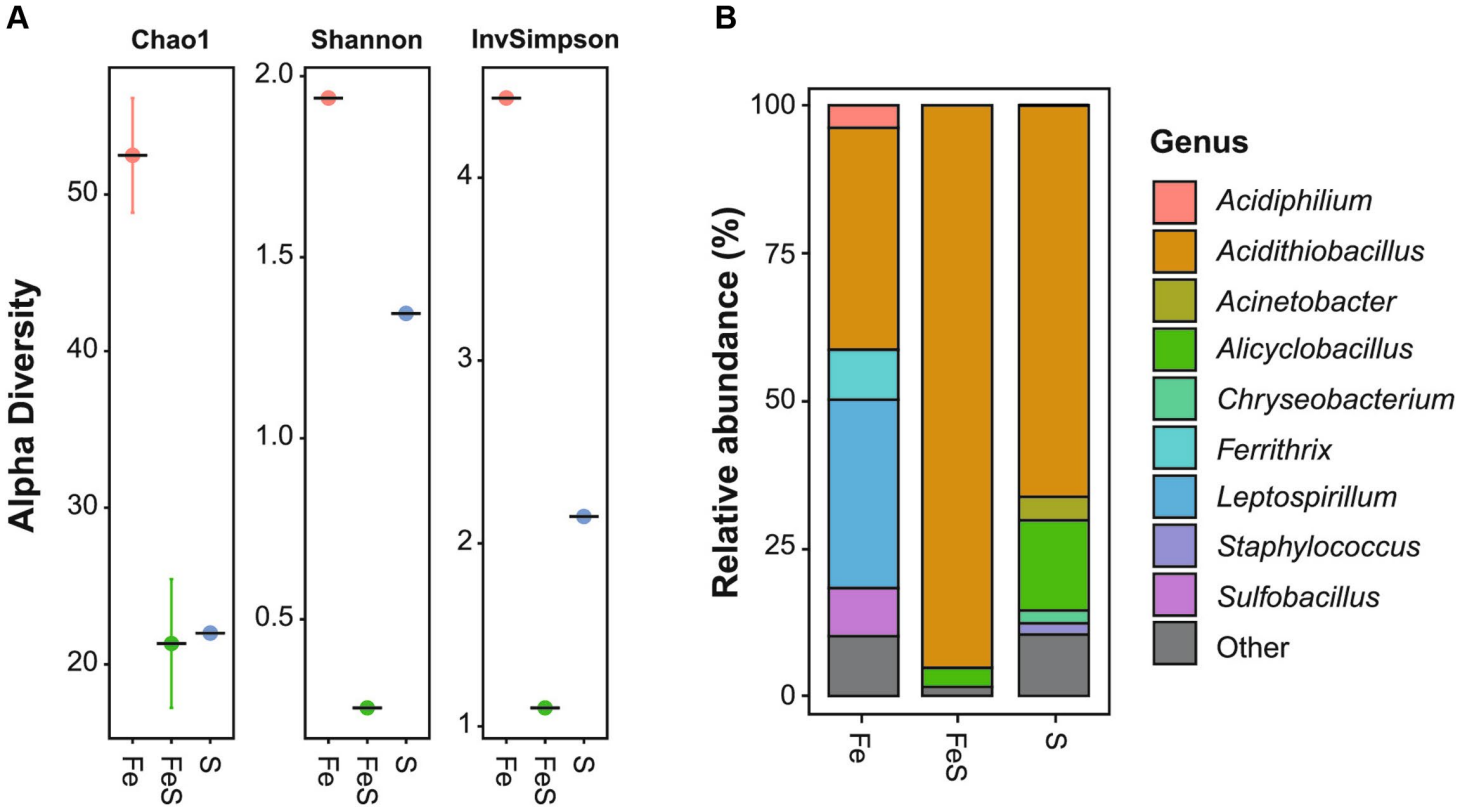
Microbial enrichment of acidic mine pit lake sediment in a selective medium for isolating acidophiles supplemented with Fe^2+^ (red circle; Fe), S^0^ (blue circle; S), and both electron donors (green circle; FeS). Amplicon sequence variants richness **(A)** and relative abundance **(B)** of the ten most abundant genera. Detailed information is given in Supplementary Table S2.

The highest microbial diversity was observed in the enriched culture in Fe medium (Figure 1A), with the dominant genera being *Acidithiobacillus* (38%), out of which *A. thiooxidans* and *A. ferrooxidans* accounted for 33 and 5%, respectively, and *Leptospirillum* (32%), out of which *L. ferrooxidans* accounted for the majority. In addition, other genera such as *Ferrithrix* (8%), *Sulfobacillus* (8%), and *Acidiphilium* (4%) were identified with lower relative abundance (Figure 1B). The enriched culture in S medium was found to be dominated by *A. thiooxidans* (66%) and *Alicyclobacillus* (15%), with the majority of the latter being *Alicyclobacillus* (*Acb.*) *disulfidooxidans*. In addition, *Acinetobacter* (4%), *Chryseobacterium* (2%), and *Staphylococcus* (2%) were identified (Figure 1B). The lowest microbial diversity was observed in the enriched culture in FeS medium (Figure 1A), which was highly dominated by *A. thiooxidans* (95%), followed by *Alicyclobacillus* (3%), with *Acb. disulfidooxidans* constituting the majority of the genus abundance (Figure 1B). Mainly, acidophilic Fe^2+^- and RISC-oxidizing chemoautolithotrophs were detected in the enriched cultures, which was consistent with the selective media being of low pH and containing only inorganic electron donors and no organic C source. The extreme acidophile *A. thiooxidans* can utilize RISCs such as S^0^, thiosulfate, and tetrathionate as sole electron donors but cannot oxidize Fe^2+^. *L. ferrooxidans* can utilize only Fe^2+^ as an electron donor (Hippe, 2000). On the other hand, *A. ferrooxidans* and *Acb. disulfidooxidans* (formerly *Sb. thermotolerans*) can oxidize both RISCs and Fe^2+^ (Kelly and Wood, 2000; Karavaiko et al., 2005).

**Figure 2:**
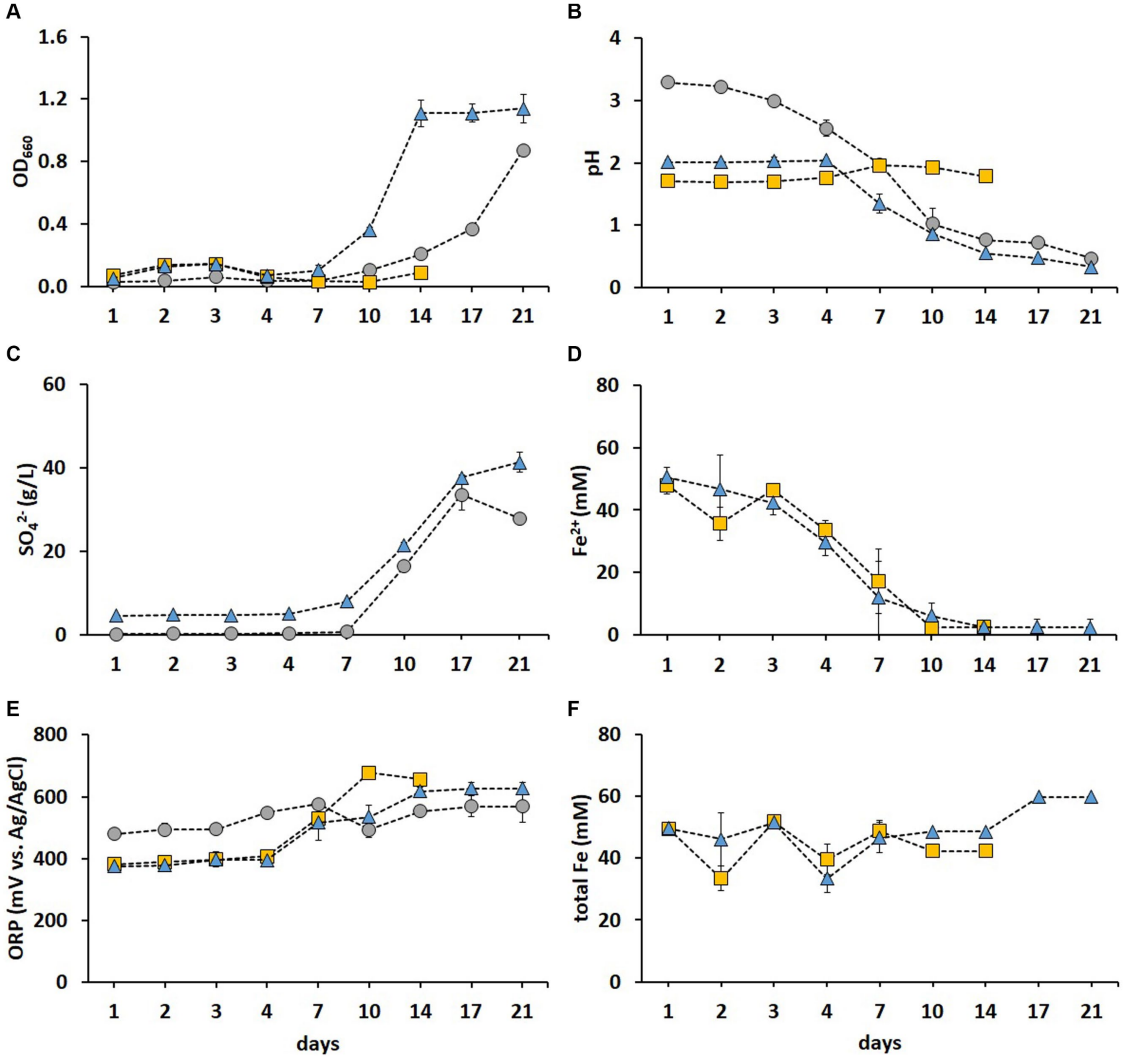
Time course of OD_660_ **(A)**, pH **(B)**, SO_4_^2−^ concentration **(C)**, Fe^2+^ concentration **(D)**, ORP **(E)**, and total Fe concentration **(F)** during the microbial enrichment in a selective medium for isolating acidophiles supplemented with S^0^ (grey circle), Fe^2+^ (gold square), and both electron donors (blue triangle). Standard deviations (*n* = 3) are indicated with vertical bars and, if not shown, are smaller than the size of the symbol.

The cell growth was most pronounced in the FeS medium (Figure 2A), while pH decreased drastically after day 7 in both S and FeS media, resulting in final pH values of 0.47 and 0.33, respectively (Figure 2B). Correspondingly, the SO_4_^2−^ concentration increased in S and FeS media after day 7 (Figure 2C). The above substantial decrease in pH and increase in SO_4_^2^ resulted from S^0^ oxidation catalyzed by RISC oxidizers such as *A. thiooxidans* and *Acb. disulfidooxidans* (Dopson and Johnson, 2012). *A. thiooxidans* and *Acb. disulfidooxidans* have both been detected in industrial bioleaching heaps processing copper sulfides, with a stable abundance of *A. thiooxidans* throughout different phases of the leaching process (Remonsellez et al., 2009). Thus, these enrichments provided promising species for the following adaptive stages prior to bioleaching. The Fe^2+^ was fully oxidized after day 10 in Fe and FeS media (Figure 2D), which resulted in ORP exceeding +600 mV (Figure 2E), in agreement with Hansford and Vargas (2001). *A. ferrooxidans* and *L. ferrooxidans* were mainly responsible for Fe^2+^ oxidation in Fe medium, while *Acb. disulfidooxidans* oxidized Fe^2+^ in FeS medium. As previously reported (Ishigaki et al., 2005), the metal recovery rate in fly ash bioleaching can be significantly improved by using mixed cultures of Fe^2+^ and RISC oxidizers as opposed to pure cultures. Similarly, the recovery rates of Zn, Co, Cu, and Mn from incineration residues increased from 50% achieved with pure cultures to nearly 100% when a mixed culture of RISC and Fe^2+^ oxidizers was used (Kremser et al., 2021). The Fe present in the sediment was partly solubilized after day 10 in FeS medium which is depicted by the total Fe concentration increasing above 50 mM (Figure 2F). In the present study, the enriched culture in FeS medium exhibited the highest growth and oxidation rates among the three enrichments tested and was thus chosen for further metal adaptation and two-step BM bioleaching. Moreover, this culture showed the lowest microbial diversity, thereby providing species stability during the successive processes.

### 3.2 Adaptation to elevated metal concentrations

**Figure 3:**
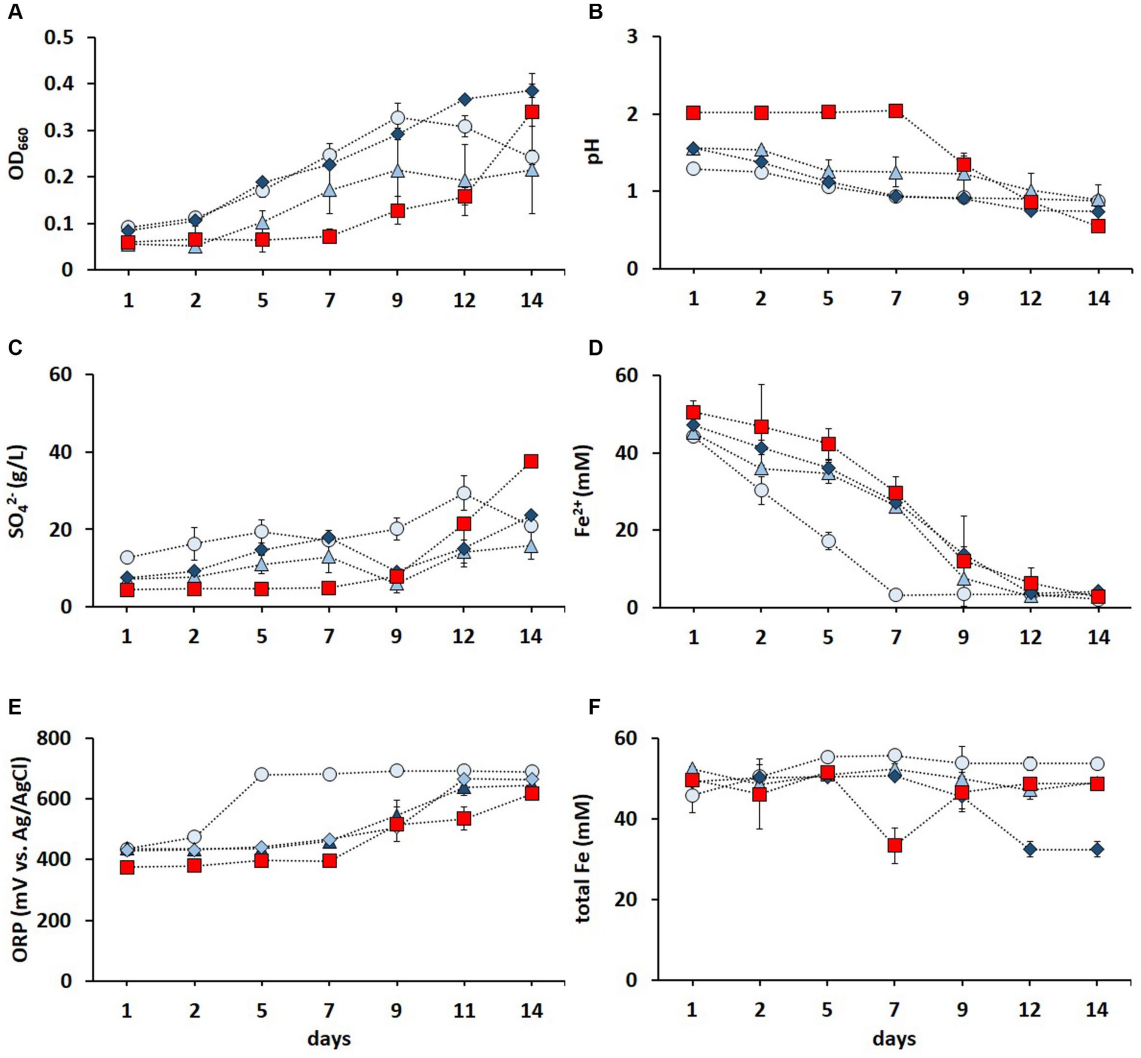
Time course of OD_660_ **(A)**, pH **(B)**, SO_4_^2−^ concentration **(C)**, Fe^2+^ concentration **(D)**, ORP **(E)**, and total Fe concentration **(F)** in non-adapted (red square) and adapted enriched culture in the FeS medium during the first (light blue circle), second (blue triangle), and third (dark blue diamond) adaptive stage to elevated concentrations of Li^+^, Co^2+^, Ni^2+^, Mn^2+^, and Cu^2+^. Standard deviations (*n* = 3) are indicated with vertical bars and, if not shown, are smaller than the size of the symbol.

Contact bioleaching of LIBs can be challenging due to the potential inhibition of acidophiles caused by the acid-consuming nature of LIBs and high dissolved metal concentrations (Roy et al., 2021b). Mixed cultures of Fe^2+^ and RISC oxidizers have been shown to be more resilient than pure cultures (Qiu et al., 2005; Akcil et al., 2007). The metal tolerance of the enriched culture in FeS medium was improved via three subsequent adaptive stages (Figure 3). As shown in Figure 3A, increasing metal concentrations did not hamper the cell growth. On the contrary, the OD_660_ values were higher during all three adaptative stages (until day 12) than those in the non-adapted cultures. The decrease in pH (Figure 3B) and increase in SO_4_^2−^ concentration (Figure 3C) confirmed the activity of RISC-oxidizing microorganisms during the adaptive stages. After 2 weeks, all cultures oxidized Fe^2+^ to Fe^3+^, but the culture in the first adaptive stage completely oxidized Fe^2+^ after 1 week, while the elevated metal concentrations in the second and third adaptive stages reduced the Fe^2+^ oxidation rate compared to the first stage but not to the non-adapted culture (Figure 3D). A similar trend was also observed in the ORP values (Figure 3E).

### 3.3 Two-step bioleaching of LIBs

**Figure 4:**
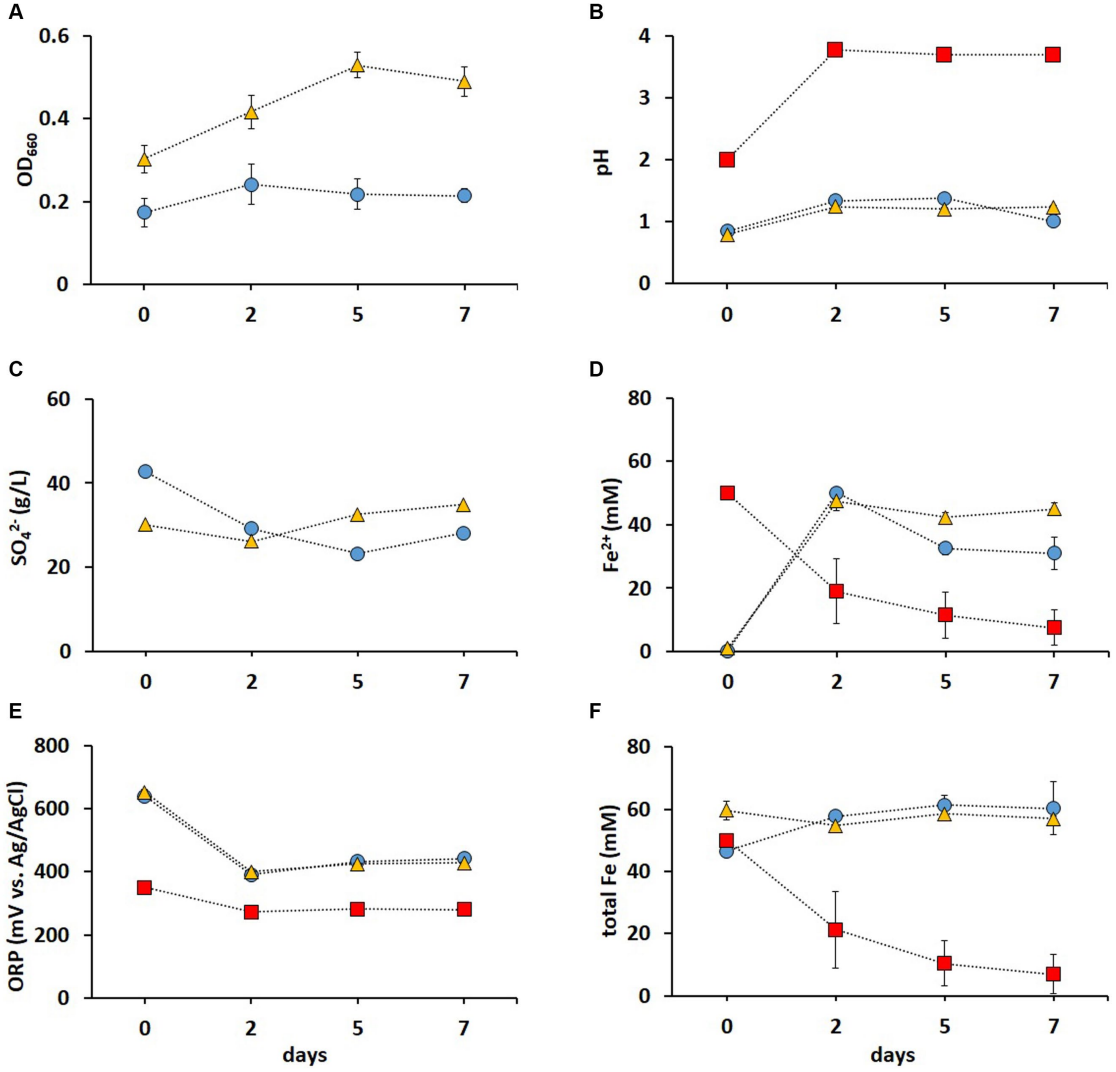
Time course of OD*_660_* **(A)**, pH **(B)**, SO_4_^2−^ concentration **(C)**, Fe^2+^concentration **(D)**, *ORP* **(E)**, and total Fe concentration **(F)** during second step of direct bioleaching of 1% (w/v) pulp density BM using non-adapted (blue circle) and adapted (gold triangle) enriched cultures in FeS medium, and control abiotic FeS medium (red square). Day 0 indicates the end of the 7 days pre-cultivation of the leaching cultures and the addition of BM. Standard deviations (*n* = 3) are indicated with vertical bars and, if not shown, are smaller than the size of the symbol.

It has been previously shown that contact bioleaching of high loads of LIBs can lower metal recovery rates (Niu et al., 2014). Therefore, two-step bioleaching of metals from 1% (w/v) pulp density BM was investigated in the present study, using the non-adapted and adapted enriched cultures in FeS media described above (Figure 3). As shown in Figure 4A, the adapted cultures showed higher OD_660_ values than those of the non-adapted cultures, indicating a positive effect of the adaptation on the cell growth. In most studies investigating bioleaching of LIBs, pH adjustments were required due to the acid consumption by the alkaline source material. Heydarian et al. (2018) reported that adapted mixed cultures did not grow above 4% (w/v) pulp density until the pH was lowered. However, pH remained below 1.5 in both non-adapted and adapted cultures throughout the whole leaching experiment in this study (Figure 4B), which enhanced the metal release and prevented potential Fe precipitation [Fe^3+^ typically precipitates at pH > 2 (Nurmi et al., 2010)]. The pH in the abiotic control increased to pH > 3 after 2 days (Figure 4B), which was attributed to the alkaline nature of BM (Wood et al., 2020). A gradual adaptation (from 1 to 5% chalcopyrite) improved the resistance of *A. ferrooxidans* to Cu during bioleaching of the mineral, resulting in a shorter lag phase in an adapted culture compared to that in a non-adapted one (Xia et al., 2008). In this study, the adapted cultures reached higher OD_660_ values (Figure 4A) and SO_4_^2−^ concentration (Figure 4C) compared to the non-adapted cultures, indicating that the adaptation to elevated metal concentrations improved the growth and oxidation rates during BM bioleaching. Almost all Fe^2+^ was oxidized to Fe^3+^ at the end of pre-cultivation (day 0), and this was partially reduced back to Fe^2+^ during the BM bioleaching phase on days 2–7 in biotic experiments (Figure 4D). The ORP was around +400 mV in both non-adapted and adapted cultures (Figure 4E), correlating with Fe speciation (Figures 4D,F). In addition, the pH increase resulted in precipitation of Fe, indicated by the decrease in total Fe concentration in solution (Figure 4F). In contrast, no Fe precipitation occurred when bacterial cultures were used; the bacteria maintained a low pH of around 1 and total Fe around 50 mM, further supporting Fe^2+^/Fe^3+^ cycling.

**Figure 5:**
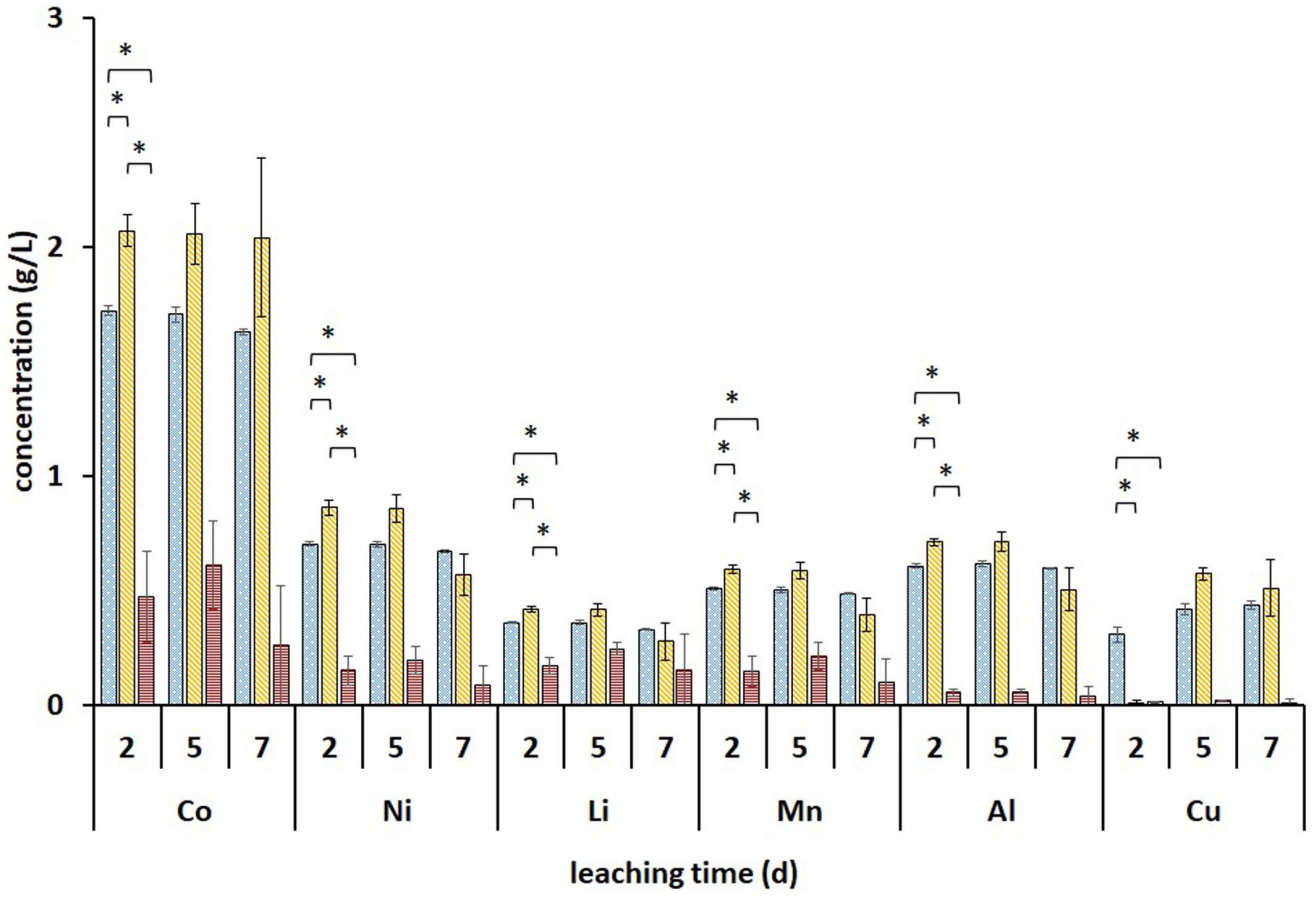
Changes in the concentrations of solubilized metals during two-step direct bioleaching of 1% (w/v) of BM using non-adapted (blue dotted) and adapted (gold diagonal stripes) enriched cultures in FeS medium compared to control abiotic FeS medium (red horizontal stripes). An asterisk indicates a significant change (*p* < 0.05). Standard deviations (*n* = 3) are indicated with vertical bars and, if not shown, are smaller than the size of the symbol.

Metal analysis of leachates collected on days 2, 5, and 7 from the (bio)leaching experiments showed that the maximum concentrations of dissolved Li, Co, Ni, Mn, and Al were obtained on day two and did not increase further (Figure 5). During these 2 days, all Fe^3+^ was reduced in non-adapted and adapted cultures (Figure 4D). This indicates that mainly biogenic lixiviants (containing H_2_SO_4_ and Fe^3+^) produced by the microorganisms during pre-cultivation were involved in the metal leaching. Complete oxidation of Fe^2+^ in abiotic FeS medium was achieved after 7 days (Figure 4D), indicating that chemical oxidation of Fe^2+^ by metal oxides in BM is relatively slow in acidic environments. On the other hand, the Fe^2+^ concentration in the enriched cultures decreased slightly from day 2 to 7, which may imply that besides Fe^2+^ oxidation, either chemically or by *Alicyclobacillus*, aerobic Fe^3+^ reduction also occurred in the presence of *A. thiooxidans*. In this case, Fe^3+^ was probably reduced by the RISC intermediates produced during bacterial S^0^ oxidation (Breuker and Schippers, 2023). Thus, iron cycling during BM bioleaching appears to be influenced by both abiotic and biotic reactions.

**Figure 6:**
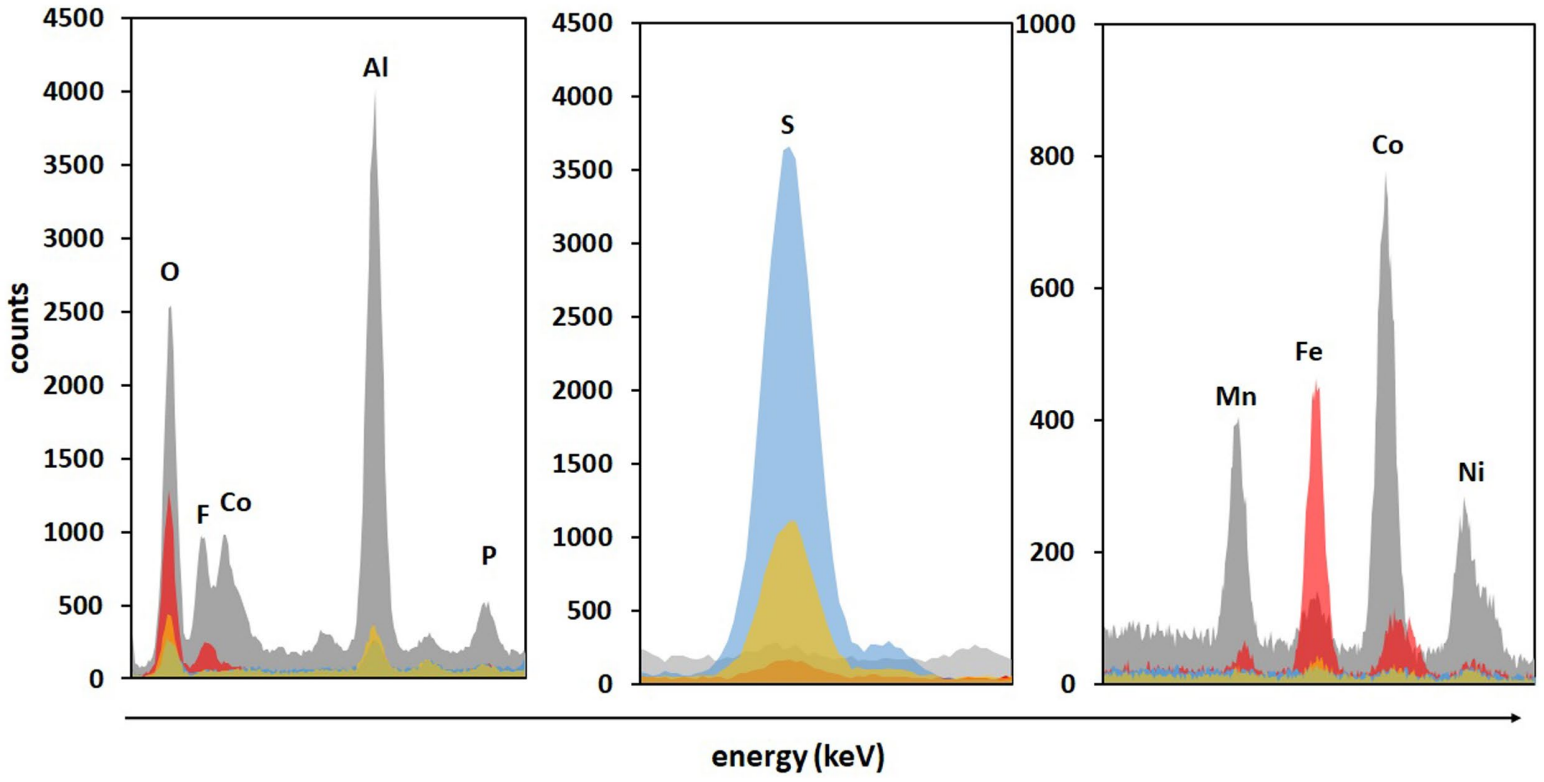
The SEM/EDS analysis of BM before (grey color) and after two-step direct (bio)leaching with non-adapted (blue color) and adapted (gold color) enriched cultures in FeS medium compared to control abiotic FeS medium (red color).

Significantly higher concentrations of all monitored metals (except Cu) were detected in adapted cultures on day 2 compared to those in non-adapted cultures and abiotic control. Additionally, solid residues were analyzed using SEM/EDS. Figure 6 shows that almost all Co, Ni, Mn, and Al were leached from the BM in non-adapted and adapted cultures. Abiotic control was slightly less efficient in extracting Co and Mn. The high S signals in both non-adapted and adapted cultures can be attributed to the residual electron donor added to the medium (Figure 6). The difference between the two intensities indicates that in the presence of BM, the adapted cultures oxidized S^0^ more effectively than the non-adapted cultures, which is also evident from SO_4_^2−^ concentrations (Figure 4C). Similarly, Hosseini et al. (2022) reported enhanced SO_4_^2−^ production and Sr. and Ce recovery during bioleaching of gold mine tailings when *A. thiooxidans* adapted to 4% (w/v) tailings pulp density was used. The Fe precipitation in the abiotic assay due to the increase in pH and the absence of Fe^2+^ and RISC oxidizers was confirmed by the high Fe signal in the solid residue (Figure 6).

The Li leaching is thought to be primarily due to dissolution in H_2_SO_4_, while the dissolution of other metals such as Co and Ni occurs by a combination of acidolysis and redoxolysis via Fe^2+^/Fe^3+^ cycling (see Eq. 1) (Xin et al., 2009).


(1) $$\begin{array}{l} 2{\mathrm{FeSO}}_{4}+2{\mathrm{LiCoO}}_{2}+4H_{2}{\mathrm{SO}}_{4}\to {Fe}_{2}{\left({\mathrm{SO}}_{4}\right)}_{3}+2{\mathrm{CoSO}}_{4}\\ +{Li}_{2}{\mathrm{SO}}_{4}+4H_{2}O \end{array}$$

Co and Ni are predominantly present in LIBs in the oxidation state +3, which are less soluble than their reduced state +2. Wu et al. (2019) showed that the presence of pyrite and Fe^2+^ enhanced the efficiency of Co and Ni bioleaching, with Fe^2+^ reducing Co^3+^ and Ni^3+^ to Co^2+^ and Ni^2+^. Moreover, the presence of Ag^+^ promoted the dissolution of Co from LiCoO_2_ by bioleaching with *A. ferrooxidans* via formation of AgCoO as an intermediate (Zeng et al., 2013). Zeng et al. (2012) reported an increase in Co extraction from 43.1 to 99.9% during bioleaching in the presence of Cu^2+^ as a catalyst (Eqs 2, 3).


(2) $${Cu}^{2+}+2{\mathrm{LiCoO}}_{2}\to {\mathrm{CuCo}}_{2}O_{4}+{2Li}^{+}$$

(3) $${\mathrm{CuCo}}_{2}O_{4}+{6Fe}^{3+}\to {6Fe}^{2+}+{Cu}^{2+}+2O_{2}+{2Co}^{2+}$$

In this study, it is presumed that Li was leached by H_2_SO_4_ generated by S^0^ oxidation using the enriched culture dominated by *A. thiooxidans* and *Acb. disulfidooxidans*, while Co, Ni, and Mn were likely solubilized by the combination of acid dissolution and reduction by Fe^2+^. Still, at low pH, chemical oxidation of Fe^2+^ to Fe^3+^ competes with bacterial oxidation (in this study by *Alicyclobacillus*). It appears that Fe^3+^ participates in the dissolution of the material along with H_2_SO_4_, and the Fe^2+^ produced may participate in the reduction of other metals or serve as an electron donor for bacterial oxidation. The resulting re-oxidized Fe^3+^ might dissolve the material further or can be reduced by RISC intermediates during bacterial S^0^ oxidation. Lower pH (< 1.5) and higher ORP (> 400 mV) presented favorable conditions for the leaching of metals such as Co, which is in agreement with a study by Li et al. (2013b) who reported that the dissolution of Co from LiCoO_2_ by *A. ferrooxidans* was highly dependent on ORP with best results obtained at pH 1.5 and ORP > 400 mV measured using Ag/AgCl as reference electrode (which is similar to the electrode used in this study). Furthermore, BM contains a high proportion of Cu, which can dissolve and act as a catalyst via the formation of intermediates such as CuCo_2_O_4_, which further promotes the dissolution of Co/Ni/Mn.

## 4 Conclusion

A mixed culture of *A. thiooxidans* and *Acb. disulfidooxidans* enriched from a sediment sample collected from an acidic mine pit lake showed promising results during bioleaching of metals from spent NMC-based BM. The microbial performance was enhanced by adaptation carried out with synthetic polymetallic solutions, which reduced the stress caused by the alkaline character of BM. The mixed metal concentration was increased in three consecutive steps up to concentrations corresponding to 1% BM pulp density. During two-step bioleaching with the adapted microbial consortium, high metal leaching efficiencies were achieved. Up to 100% of Li, Co, Ni, Mn, and Al was solubilized by the combined effect of biogenic H_2_SO_4_ and Fe^3+^, together with chemical reduction of metal oxides in BM by Fe^2+^, indicating that both acid production and iron cycling play important roles in BM bioleaching. In addition, Cu released from BM likely acted as a catalyst, further improving metal dissolution. The current study shows that microbial adaptation and selection of suitable process parameters can improve bioleaching performance. Nevertheless, further research is needed to assess the effect of higher BM concentrations, before the biotechnology can be considered an economically feasible process.

## Data availability statement

The datasets presented in this study can be found in online repositories. The names of the repository/repositories and accession number(s) can be found in the article/Supplementary material.

## Author contributions

LL: Data curation, Formal analysis, Investigation, Visualization, Writing – original draft, Conceptualization. JK: Data curation, Formal analysis, Investigation, Resources, Visualization, Writing – review & editing. WR: Data curation, Formal analysis, Writing – review & editing. EP: Supervision, Writing – review & editing. DS: Resources, Writing – review & editing. MM: Writing – review & editing, Funding acquisition, Resources. KK: Conceptualization, Funding acquisition, Methodology, Project administration, Supervision, Validation, Visualization, Writing – review & editing. GG: Funding acquisition, Resources, Supervision, Writing – review & editing.
